# The promise of telemedicine in Pakistan: A systematic review

**DOI:** 10.1002/hsr2.438

**Published:** 2022-01-07

**Authors:** Syed Sarosh Mahdi, Raheel Allana, Gopi Battineni, Tamsal Khalid, Daniyal Agha, Mariam Khawaja, Francesco Amenta

**Affiliations:** ^1^ Department of Community Dentistry Jinnah Medical and Dental College, Sohail University Karachi Pakistan; ^2^ Athena Center for Advanced Research in Healthcare Camerino Italy; ^3^ Center of Clinical Research, Telemedicine & Telepharmacy Department School of Medicinal and Health products Sciences, University of Camerino Macerata Italy; ^4^ Department of Pediatrics & Child Health Aga Khan University Hospital Karachi Pakistan

**Keywords:** mHealth, remote areas, telehealth, telemedicine, web‐based systems

## Abstract

**Background:**

Telemedicine offers the possibility of provision of medical assistance to remote patients, and it has great potential in developing countries like Pakistan. Telemedicine solves logistical barriers, gives support to weak health systems, and helps to establish worldwide networks of healthcare professionals. Because of the high implementation costs, it is not possible yet to adopt telehealth systems for low‐ and middle‐income nations.

**Objective:**

To present a revision of region‐based telemedical services in Pakistan.

**Methods:**

Libraries such as PubMed (Medline), CINAHL (Cumulative Index to Nursing and Allied Health Literature), Scopus (EMBASE), and Google Scholar were used for document search. Newcastle‐Ottawa Scale (NOS) was adopted to conduct study quality. Many of the studies (n−8) included in the review were of high quality as assessed through the Newcastle‐Ottawa scale. Selected study characteristics were further analyzed based on different parameters such as publication year, sample size, study design, methods, motivation, and outcomes.

**Results:**

Search produced 955 articles and 11 items were ultimately selected to conduct the review. These studies were further characterized as region‐based telemedicine implementation. Out of 11, eight studies were conducted in the urban region and three studies were conducted in the rural areas of Pakistan. Many studies produced evidence on telehealth interventions by smartphone services such as SMS, apps, and web‐based telemedicine.

**Conclusions:**

Telehealth interventions such as mHealth, eHealth, telemedicine, and telepharmacy in Pakistan were introduced starting from the last two decades. For obtaining the full benefits of these technologies, it is necessary that they but certainly need to become an integral part of Pakistan's current health infrastructure.

## 1 INTRODUCTION

Telemedicine is an integration of technology and medicine.^1^ Telemedicine is the way to provide better health care to people in isolated, difficult access areas or zones suffering from a shortage of health services.^2^ One of the objectives of telemedicine is to provide equal access to medical expertise irrespective of the geographical location of the person in need. It is more efficacious and extremely useful especially where experts are rare, distances are large, and/or infrastructure is limited.^3^

The first experiences of telemedicine date at the beginning of the previous century, when in 1903, Einthoven transmitted the first electrocardiogram. Radiotelegraphy was also used from 1920 for assisting patients onboard ships, which represent the prototype of remote and isolated places.^4^ Modern telemedicine techniques have been underdevelopment for nearly 50 years by Wittson and colleagues, which were the first to employ IATV for medical purposes.^5^ In 1959, they used a microwave link for telepsychiatry consultations between the Nebraska Psychiatric Institute in Omaha and the state mental hospital 112 miles away. In the 1970 and 1980s, limited telemedicine projects were instituted at several sites in North America and Australia, including the Space Technology Applied to Rural Papa go Advanced Health Care (STARPAHC) project of the National Aeronautics and Space Administration (NASA) in southern Arizona, a project at Logan Airport in Boston, Mass, and programs in northern Canada.^6^ Except for the assistance to ships with no medical facilities onboard, which are now assisted by specialized institutions called Telemedical Maritime Assistance Services (TMAS), a 20‐year‐old telemedicine program at the Memorial University of Newfoundland, St‐John's, none of the programs born before 1986 has survived. Although data are limited, the early reviews and evaluations of those programs suggest that the equipment was reasonably effective at transmitting the information needed for most clinical uses and that users were, for the most part, satisfied.^7^

Telemedicine has a strong impingement in developing countries since it allows remote areas of a country to get access to medical care and to create local knowledge.^8^ It is a flourishing industry, valued at billions of dollars globally.^9^ It has been of particular interest for the past few years due to its ability to transcend the common barriers, which prevent people from accessing health care.^10^ These include long distances to a functional health facility, lack of doctors in rural areas, and the high cumulative costs associated with a doctor's visit (cost of transportation, income lost due to time off work, and the doctor's fee).

Telemedicine holds the promise of being able to connect patients in the remotest of regions to qualified doctors in urban areas. Pakistan, like most emerging markets, has a healthcare problem. The rapid increase in population combined with an unstructured system of health care has led to an uneven distribution of doctors, which results in the chronic shortage of doctors in peri‐urban and rural areas. In 2012, there were 0.8 doctors per population of 1000 residents. Like in other emerging markets, there is neglect for healthcare in general, with Pakistan having spent only 0.9% of its GDP on healthcare in 2014, which was the lowest in all of Asia.^9^ Approximately 61% of the Pakistani population lives in rural areas. Realizing all the promise that telemedicine holds in revolutionizing health care in emerging markets, the governments of India, Bangladesh, Kenya, and Uganda, among others have implemented telemedicine programs that are providing health care through either audio, video, text messages, and different applications (apps).

These programs have revolved around maternal and child health, pregnancy, prevention, and diagnosis of acquired immunodeficiency syndrome (AIDS), etc. Sub‐Saharan Africa, for example, implemented 487 unique telemedicine programs from 2006 to 2016.^11^ In more advanced markets, like India, companies are now beginning to develop chatbots, using artificial intelligence (AI) to solve common healthcare problems. With solutions of this type, patients do not need to visit the doctor for minor ailments at all.^12^ With so much exciting news coming from emerging markets, one must ask the question, where does Pakistan stand in all this? According to the World Health Organization (WHO) telemedicine survey of 2016, Pakistan has no telemedicine laws or regulations in place.^9^

Telemedicine has obvious advantages in the case of emergencies in remote environments such as on ships, in planes, and possibly on the battlefield.^10^ In all these situations, it is very difficult that patients can be referred to the doctor in time. Telemedicine has and continues to benefit the Pakistani people healthcare system in terms of preventive care and disease treatment. Several companies are in the process of providing the telecommunication support needed for telemedicine, but much remains to be accomplished before telemedicine can glean its bragged benefits for Pakistan's exponentially growing population. Pakistan is in a unique position for building its telemedicine infrastructure. With its highly qualified medical practitioners and an emerging technological industry, the country can create products and services to cater to this evolving area. Given proper access and awareness, Pakistan seems Equanimeous to incorporate telemedicine beyond its current rudimentary projects to large‐scale programs that can serve as a model for itself and the developing world.^2^

By considering these facts, we have reviewed the current situation regarding telemedicine's awareness, implementation, and current trends in Pakistan through a systematic analysis of published studies in the last 20 years. The review aimed at understanding the current penetration, accessibility, knowledge, barriers, and areas of interest with regard to the use of telemedicine‐based interventions in urban and rural Pakistan. This can help public authorities in better understanding the diverse bottlenecks in telemedicine sustainability and execution, to propose clear measures and problem prioritization. The rest of the article is articulated as follows. In Section 2, presents methods that incorporate in study collection, quality assessment, and inclusion and exclusion criteria. Section 3 presents the search outcomes and overview of the study results and Section 4 provides the discussion on the main results of the current research. Section 5 ends up with study conclusions.

## 2 METHODOLOGY

### 2.1 Document search

A systematic analysis of the literature was performed by collecting data from the PubMed (Medline), CINAHL (Cumulative Index to Nursing and Allied Health Literature), EMBASE, and Google Scholar repositories. The last date search was November 20, 2020, to identify relevant studies. For our systematic analysis, the PRISMA literature quest guidelines were followed. The key search terms included (“Telemedicine” OR “Telecare” OR “Mobile health” OR “eHealth” OR “mHealth” OR “e‐health” OR “telehealth”) and (“Pakistan”). The sources of each qualifying paper were also scanned for related papers that may not have appeared in the search term results.

**FIGURE 1:**
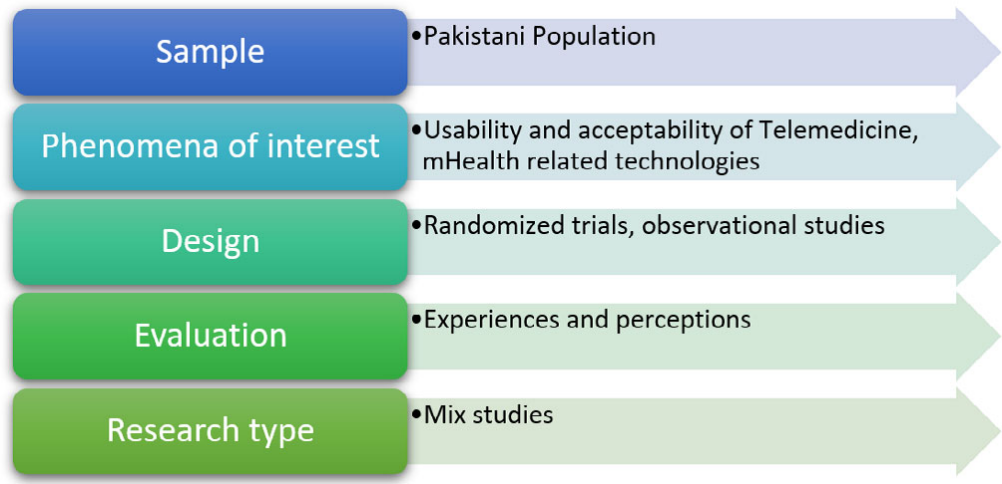
Spider strategy used in the research analysis

The search and selection of studies followed the SPIDER question format and the PICO tool (Figure 1). Experts were also contacted to find any studies that were missing. Two reviewers among coauthors independently checked the title/abstracts of all the studies listed for inclusion. The full text of the papers was retrieved for further assessment of potentially qualifying research and two review authors separately tested their eligibility. Possible differences were settled through negotiation with a third review author. References to full‐text publications meeting the inclusion criteria were reviewed to identify other related records. No grey literature has been searched or included in the study, as well as no dissertations or unpublished studies.

### 2.2 Inclusion/exclusion criteria

The eligibility criteria for the analysis were that the research should be in the English language. No criteria were set up for the range of the studies and the explanation for this is that only a few studies were identified for mHealth or telehealth in an LMIC setup like Pakistan. The demographic limitation was set up as we included only those studies that have been conducted in Pakistan. There were no limitations on the study design except for reviews, short communications, or editorials. No limitations on the area of research were applied, although due to the scope of the analysis, most could be categorized as mHealth, eHealth, or telehealth.

Exclusion criteria included narrative reviews, short communications, editorials, and letters to the editors. Telehealth or mHealth studies that were conducted in other low‐ and middle‐income countries (LMICs) setup outside Pakistan were excluded. Studies published in non‐indexed or non‐peer‐reviewed journals were also excluded.

### 2.3 Quality assessment

Quality assessment was conducted in two phases. As the first step, after having excluded irrelevant items, remained full‐text eligible articles. These were distributed equally among authors for further reading. Each paper was verified for quality check according to the Newcastle‐Ottawa Scale (NOS) which ranges from 0 to 9. The scaling factor was decided considering different parameters such as study selection, preliminary outcomes, and comparability. Based on study quality resulting from the results of the NOS scoring, papers were divided into three categories, namely poor (0‐4), moderate (5‐6), and good (7‐9) quality. The authors collectively decided these scores by recording each quality parameter score on independent excel sheets. The majority of the studies included in the final review achieved a good (NOS ≥ 7) rating as detailed in Table 1. Selected publications were further characterized based on publication year, sample size, study design, methods, motivation, and outcomes that were further explained in the next section.

**TABLE 1:** Newcastle‐Ottawa scale quality assessment for nonrandomized studies considered for the review

	Selection				Comparability		Outcome			Overall
Study/reference	1	2	3	4	5	6	7	8	9	Score (*)
Gul et al^13^	*	*		*	*		*	*	*	7
Siddiqui et al^14^	*	*		*	*	*	*		*	7
Kazi et al^15^	*	*	*	*	*	*	*	*	*	9
Kazi et al^16^	*	*	*		*	*	*	*	*	8
Sayani et al^17^		*	*		*	*	*	*	*	7
Iftikhar et al^18^	*	*	*		*	*	*	*	*	8
Akbar et al^19^	*	*	*		*	*	*		*	7
Kamal et al^20^	*	*	*	*	*	*	*	*	*	9
Ashfaq et al^21^	*		*		*	*	*	*	*	7
Zaidi et al^22^	*		*	*	*	*	*		*	7
Tariq et al^23^	*	*			*	*	*	*	*	7

*Note*: Quality of studies: Poor (0–4*), Moderate (5–6*) Good (7–9*).

* Category scoring criteria selection, maximum score: 5 stars, low selection bias (1 star); Acknowledged selection bias (1 star); Highly selected group (0 stars); No description (0 stars); Selection of non‐exposed group (maximum 1 star); Source population as exposed group (1 star); Drawn from different source (0 stars); No description (0 stars); Ascertainment of exposure (maximum 1 star); Secure record (eg, pathological record) (1 star); Structured interview (1 star); Written self‐report (0 stars); No description (0 stars); Outcome demonstrably absent at baseline (maximum 1 star); Yes (2 stars), No (0 stars); Comparability: (maximum 2 stars); Comparability of cohorts, controlling for confounders (maximum score: 2 stars); Controls for key confounders (eg, Gleason grade, 1 star); Controls for related factors (1 star); Cohorts/confounders (incomparable/uncontrolled, 0 stars); Outcome assessment (maximum score: 3 stars); Large studies/panels, secured records or directly measured (1 star); Self‐reported information (0 stars); Single target/objective (0 stars); Adjusted for missing data of follow‐up (1 star); No follow‐up or statement about missing data (0 star). Clear specification of outcomes (Yes: 1 star, No: 0 stars).

## 3 RESULTS

### 3.1 Search outcomes

**FIGURE 2:**
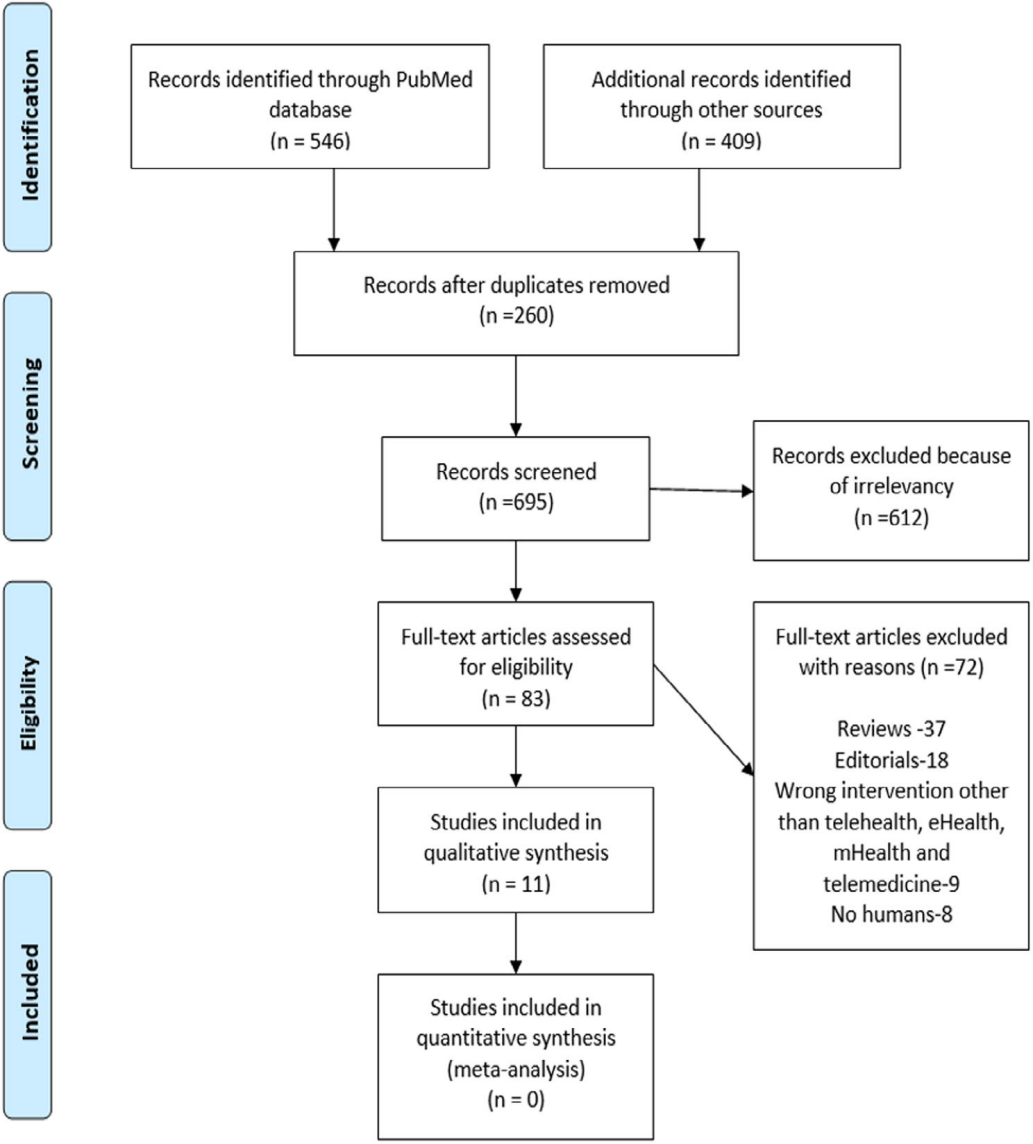
Systematic review study selection approach by PRISMA^24^

Figure 2 (PRISMA chart) summarizes the results of the study. Articles were comprehensively reviewed for eligibility. A total of 955 articles were retrieved from different databases mentioned above. A total of 260 duplicates were removed after search results to an endnote. A total of 83 studies remained for full‐text review after excluding 612 irrelevant studies.

### 3.2 Study characteristics

Table 2 summarizes the characteristics of the 11 selected studies after quality assessment. All the studies included were conducted in Pakistan. Out of 11, eight studies were conducted in the urban region,^13^, ^14^, ^15^, ^16^, ^18^, ^20^, ^21^, ^23^ whereas three studies were conducted in the rural areas of Pakistan.^17^, ^19^, ^22^ Most of the studies were of the cross‐sectional type,^13^, ^14^, ^17^, ^18^, ^21^, ^23^ followed in the descending order by randomized control trials,^15^, ^16^, ^20^ quasi‐experimental design,^19^ and qualitative interview analyses.^22^

**TABLE 2:** Characteristics of the studies involved in the research

Author/reference	Study design	Sample	Methods	Motivation	Findings
Gul et al^13^	Cross‐sectional	194 paraplegics	Survey analysis	To provide skills for career applications during and after the recovery process.	The telemedicine training center was used for this purpose without special facilities or staff, thus increasing the utilization of the service.
Siddiqui et al^14^	Cross‐sectional	100 patients	Questionnaire	To assess the acceptability of mHealth	The use of phone call alerts or SMS reminders tends to be an appropriate and favorable choice for hypertensive and diabetic patients.
Kazi et al^15^	Randomized trial	3535 participants	Research Survey was conducted with WHO sampling technique and	Improving the efficacy of field‐based health professionals and health managers in tracking the immunization program.	The incorporation of the GPS into a cell phone gives it a very useful instrument for tracking and monitoring the coverage of health programmers.
Kazi et al^16^	Randomized Control Trial	356 participants	Chi‐square tests were conducted to do data analysis	Compare the proportion of infants immunized to date at 18 weeks of age.	This research assesses the enhancement of immunization coverage by SMS notifications in the HDSS community in a real‐world setting in Pakistan.
Iftikhar et al^18^	Cross‐sectional study	400 patients	Questionnaire	Evaluated the ability and desire of patients to use information technology to combat chronic diseases.	Far away patients from the healthcare center were able to use video conferencing so that they could save more than 60 min of their time.
Kamal et al^20^	Randomized Control Trial	310 stroke survivors	The analysis performed by the intention‐to‐treat principle	Determine the effect of video‐based instruction on patient and caregiver dyads after the first stroke.	Mortality among stroke patients as the number of stroke‐related complications was higher in the control group than in the intervention group and this discrepancy was statistically important (*P* < .001).
Ashfaq et al^21^	Cross‐sectional design	224 doctors	A self‐designed well‐structured questionnaire using a five‐point Likert scale	To assess knowledge of telemedicine technology among Pakistani physicians and to assess their understanding of its benefits, drawbacks, and realistic implications.	The views and expectations regarding the adoption and application of this emerging technology have been accepted by the majority of doctors, with a focus on growing understanding.
Tariq et al^23^	Cross‐sectional design	505 engineering university students	Perceived eHEALS was measured on a Likert scale and health behavior was evaluated by questionnaire.	Understand the level of internet usage for browsing health information and common health‐related topics.	The results of this work presumed eHealth awareness has not been correlated with healthy habits such as physical exercise and dietary supplement consumption.
Sayani et al^17^	Retrospective cross‐sectional study	25 182 teleconsultation visits	The *t*‐test was applied to differentiate the two‐sample homoscedastic groups. Also, a one‐way sensitivity analysis was conducted assuming personal teleconsultations at 90%, 75%, and 50%.	To project transport cost, accommodation, and the cost for teleconsultation and projected timesaving through the utilization of teleconsultation, travel time calculation.	This research examines the economic implications of telemedicine for patients in terms of cost and time savings and its possible role in improving chronic disease outcomes through data collection
Akbar et al^19^	Quasi‐experimental study	135 mothers	The data was entered EpiData (3.1) was used for data entry and IBM SPSS v21 was used to do data analysis	To evaluate the efficacy of mobile health in IYCF among pregnant and lactating mothers in Tarlai, Islamabad.	The post‐intervention survey signifies the effectiveness of mHealth in raising knowledge, attitude, and practices of mothers regarding IYCF in rural Islamabad.
Zaidi et al^22^	Qualitative interviews	26 vaccinators	Iteratively methods were used to understand the acceptability of digital technologies	To evaluate the acceptability and operability of the software for monitoring vaccine experiences within the district health department, examine the data validity and data‐related issues of stakeholders	Findings are important not only to the wide‐ranging introduction of immunization monitoring applications in Pakistan but also to inform the use of digital technology for results‐based distribution by frontline health staff.

#### 3.2.1 Telemedicine interventions in urban areas of Pakistan

As mentioned above, most studies (8/11; 73%) related to telemedicine implementation in Pakistan were focused on urban populations. One work presented the effectiveness of training rural paraplegics in telemedicine computer skills and is reported that the training and knowledge of telecommunication skills are necessary to understand telehealth usage in hospitals of Rawalpindi, Pakistan. Telehealth interventions are ranging from mobile phone outreach including short message services (SMS), mHealth‐based app, video conferencing, and web‐based telemedicine. Patients have been acquainted with the use of telemedical service websites and sending email services. Instructions included digital camera activity to collect progress images in their recovery schedule.^13^

Diabetes and hypertension are chronic diseases, and in general, the urban populations are more affected than the rural ones by these diseases. By using information technologies (IT) and mobile health (mHealth) services like SMS and call remainders, patients can reach adequate self‐management of hypertension and diabetes from home.^14^, ^18^ This will significantly enhance their self‐management and help to reduce disease complications in the future. Mobile services can also effectively help public health authorities and managers during vaccine campaigns^15^ and immunization uptake among infants.^16^ Moreover, they could promote patient data collection, analysis, and overall health management. Another video‐based education study on post‐stroke patients from the neurology ward at Aga Khan University Hospital (AKUH) in Karachi has shown the effectiveness of these programs on boosting stroke‐related mortalities and change in functional status among stroke survivors.^20^

Two cross‐sectional studies assessed telemedicine knowledge and attitude among doctors^21^ and university students.^23^ Evaluation of telemedicine use/applications among physicians was found to be in the average but doctors welcomed the integration of new technologies in Pakistan health care.^21^ University students in disciplines not related to health expressed a high confidence rate of e‐health literacy scale (eHEALS) in searching for health information on the web.^23^

#### 3.2.2 Telemedicine interventions in rural areas of Pakistan

Telehealth services are essential in rural areas to provide reasonable quality health care at low costs. This will result in benefits to rural communities of LMICs by reducing travel costs and time in accessing special care. It is reported that telemedicine has a large potential in improving chronic disease outcomes in LMICs and it can diminish the socioeconomic barriers by cost reduction, also by enabling long‐term management and early intervention.^17^

Given this, the use of mHealth interventions should be encouraged in remote areas. A study highlighted the efficiency of mHealth on young children (≤24 months). An easy‐to‐use, smartphone android‐based audiovisual application was developed for lady health workers who were then qualified and monitored to use it in the frame of the Infant and Young Child Feeding (IYCF) program.^19^ The use of these approaches enables us to understand the use of mHealth in enhancing the knowledge, practices, and attitudes of IYCF mothers in rural areas of Islamabad. Another work on childhood immunization in rural districts of Pakistan provided a qualitative experience on the utilization of digital health technologies among medical staff.^22^ The authors developed an android‐based app. The use of this app for 2 years allowed monitoring of the experience of vaccination users and district administrators and to monitor regular immunization experiences. Moreover, thanks to this app, it was possible to consider how this technology can be implemented within the vaccine delivery system. Incorporating digital health technologies on upstream health systems depends on the practice of both different stakeholders and end‐users. Recent evidence is providing positive outputs on telemedical apps development thereby creating awareness among rural communities/patients.

## 4 DISCUSSION

This article was centered on the benefits of telehealth services in developing countries like Pakistan. Telemedicine services can expand the boundaries of the practices of providers by removing the proximity barrier and by making access to health care easier. Moreover, the adoption of telemedical services during the treatment of a disease can promote changes in our idea of how to approach treatment of medical issues. The literature reviewed in this article discusses varying responses to this change. As far as I know, this is the first systematic review in 2021 summarizing the provision of telemedicine services and the diffusion of mHealth interventions in Pakistan.

### 4.1 Highlights

Increasing evidence considers telemedicine and mHealth as feasible and alternative forms of health service delivery, particularly for people living in remote areas to access health care in their local communities. Unfortunately, only a few studies have analyzed the satisfaction of patients and caregivers with telehealth. We have identified only three studies conducted in the rural region of Pakistan, suggesting that both patients and caregivers were satisfied with telehealth due to a variety of reasons.^17^, ^19^, ^25^ Specifically, attending an appointment in one's local community via telehealth compensated for the hassle of commuting long distances to an urban center for the same appointment.^17^ A few studies have used geospatial monitoring along with mHealth for the need for immunization and the significance of completing a sequence of vaccines.^15^, ^16^ One study has utilized qualitative interviews to evaluate the experience of district officials during their use of the software to track their daily immunization experience.^25^ This work has also tested the acceptability and operability of the program as a tool for tracking vaccine experience.^25^

Some studies have also considered the application of telehealth technologies in chronic conditions such as diabetes and hypertension. Iftikhar et al evaluated patients' willingness and motivation to use modern technology to tackle chronic diseases,^18^ whereas Siddiqui et al evaluated the usability and acceptability of mHealth technologies like SMS reminders to afford these chronic conditions.^14^

### 4.2 Implementation challenges

Despite technical developments, a variety of challenges are considered to affect the progress and feasibility of telehealth in rural and remote regions. These challenges include governance and stakeholder support, demonstration of economic advantage with ongoing payment capacities, adaptability to this kind of care of the target population, and effective logistical and clinical procedures.^26^ Not all communities can have enough funding for infrastructure technologies. In terms of analysis of the case for telehealth from a patient viewpoint, several trials have been performed in high‐income countries and just a few in low‐income countries.^27^, ^28^

Telemedicine operations in Pakistan are still at the primary level, even after the contribution of Elixir technology, SUPARCO, and others. There are no telemedicine services in certain parts of the country especially the remote areas except those hits by the earthquake. There is little funding for residents in rural areas to satisfy emergency care needs, such as stroke and serious injury.^2^, ^29^ The widespread reception and eventual progress of any new technology depend primarily on factors such as familiarity and appreciation of the innovation by consumers. The same is true for the skills needed for the successful application of these technologies and the working atmosphere that is conducive to their introduction. We, therefore, need studies to evaluate mHealth interventions such as SMS, automated calls, applications, and telemedicine technologies, which should be successfully incorporated into Pakistan's healthcare system.^21^

### 4.3 Possible implications

Telehealth and mHealth innovations have been complemented with the introduction and use of portable sensors and self‐care gadgets such as glucometers, mobile blood pressure controls, pulse oximeters, and optical stethoscopes in concomitance with the spreading of the COVID‐19 pandemic. The use of such technologies, as required by the pandemic, has expanded telehealth applications. A new boom in breakthrough innovations has occurred in Pakistan due to concerns of COVID‐19 contracting and social distance stress. In the last few months, social distancing has led to this technology becoming much more important as a support mechanism and to improve the use of other technologies.^30^, ^31^ The COVID‐19 pandemic presented Pakistan with an unexpected chance to solve many of the health issues by telehealth. It is time for Pakistan to discuss the use of this kind of technology in health care and to engage doctors, public health, and information technology experts to consider a variety of obstacles ahead. The implementation of policies and the establishment of adherence and monitoring mechanisms will be a major challenge. Besides the information technology network infrastructure, qualified personnel would be needed to ensure long‐term progress in telehealth and mHealth. In general, Pakistan needs a priority on health policy at all levels. With the use of information technologies, the emerging technologies could represent an enhancer necessary for a healthcare review at the government and hospital levels.^32^

### 4.4 Limitations

A main limitation of the study is in the small number of studies reviewed, which, on the other hand, have diverse and often restrictive inclusion and exclusion criteria. Most of the inclusive studies adopted similar methods such as cross‐sectional or random trials that restrict the research variety. Another limitation is the absence of quantitative studies, and this can generate knowledge gaps. The choice to exclude from this work studies published more than 15 years ago may have also missed some papers from our analysis.

## 5 CONCLUSIONS

Digital health‐based interventions such as mHealth, eHealth, telehealth, and telemedicine are increasingly becoming part of Pakistan's current health infrastructure. The advent of the COVID‐19 pandemic has created considerable potential and impetus to create innovative telehealth technologies that can improve health care and people's lives both in urban and rural regions of the country. There are already major challenges and barriers. However, amid all the obstacles, digital health continues to grow through the efforts of various stakeholders in both the public and private sectors. It is impossible to determine how successful these approaches have been, as not all interventions have been evaluated or reported. A well‐informed policy and business environment affecting both related public and private health and IT stakeholders are required to drive scale and viability for the services to be effective. A constructive cycle of policy, execution, and assessment to educate state, provincial, district, and community‐level policies would generate the platform needed to introduce more and better eHealth programs to benefit people and health workers in Pakistan.

## CONFLICT OF INTEREST

The authors declare that they have no conflicts of interest during the publication of this work.

## AUTHOR CONTRIBUTIONS

Design and Data Collection: Syed Sarosh Mahdi, Raheel Allana, Gopi Battineni

Final Manuscript Revision: Franceso Amenta

Manuscript Preparation: Syed Sarosh Mahdi, Raheel Allana, Gopi Battineni

Methods: Syed Sarosh Mahdi, Raheel Allana, Gopi Battineni

Paper Reviews: Syed Sarosh Mahdi, Raheel Allana, Gopi Battineni

Research Background: Tamsal Khalid, Mariam Khawaja, Daniyal Agha

Study Approval: Franceso Amenta

Study Planning: Syed Sarosh Mahdi, Raheel Allana, Gopi Battineni

Study Selection: Tamsal Khalid, Mariam Khawaja, Daniyal Agha

All authors have read and agreed to the published version of the manuscript.

## TRANSPARENCY STATEMENT

Dr Gopi Battineni affirms that this manuscript is an honest, accurate, and transparent account of the study being reported; that no important aspects of the study have been omitted; and that any discrepancies from the study as planned (and, if relevant, registered) have been explained.

## DATA AVAILABILTY STATEMENT

The authors confirm that the data supporting the findings of this study are available within the article.
